# Technique for sparing previously irradiated critical normal structures in salvage proton craniospinal irradiation

**DOI:** 10.1186/1748-717X-8-14

**Published:** 2013-01-12

**Authors:** Mark W McDonald, Mark R Wolanski, Joseph W Simmons, Jeffrey C Buchsbaum

**Affiliations:** 1 Department of Radiation Oncology, Indiana University School of Medicine, Indianapolis, IN, USA; 2 Indiana University Health Proton Therapy Center, Bloomington, IN, USA

**Keywords:** Proton therapy, Craniospinal irradiation, Reirradiation, Treatment planning

## Abstract

**Background:**

Cranial reirradiation is clinically appropriate in some cases but cumulative radiation dose to critical normal structures remains a practical concern. The authors developed a simple technique in 3D conformal proton craniospinal irradiation (CSI) to block organs at risk (OAR) while minimizing underdosing of adjacent target brain tissue.

**Methods:**

Two clinical cases illustrate the use of proton therapy to provide salvage CSI when a previously irradiated OAR required sparing from additional radiation dose. The prior radiation plan was coregistered to the treatment planning CT to create a planning organ at risk volume (PRV) around the OAR. Right and left lateral cranial whole brain proton apertures were created with a small block over the PRV. Then right and left lateral “inverse apertures” were generated, creating an aperture opening in the shape of the area previously blocked and blocking the area previously open. The inverse aperture opening was made one millimeter smaller than the original block to minimize the risk of dose overlap. The inverse apertures were used to irradiate the target volume lateral to the PRV, selecting a proton beam range to abut the 50% isodose line against either lateral edge of the PRV. Together, the 4 cranial proton fields created a region of complete dose avoidance around the OAR. Comparative photon treatment plans were generated with opposed lateral X-ray fields with custom blocks and coplanar intensity modulated radiation therapy optimized to avoid the PRV. Cumulative dose volume histograms were evaluated.

**Results:**

Treatment plans were developed and successfully implemented to provide sparing of previously irradiated critical normal structures while treating target brain lateral to these structures. The absence of dose overlapping during irradiation through the inverse apertures was confirmed by film. Compared to the lateral X-ray and IMRT treatment plans, the proton CSI technique improved coverage of target brain tissue while providing the least additional radiation dose to the previously irradiated OAR.

**Conclusions:**

Proton craniospinal irradiation can be adapted to provide complete sparing of previously irradiated OARs. This technique may extend the option of reirradiation to patients otherwise deemed ineligible for further radiotherapy due to prior dose to critical normal structures.

## Background

Salvage craniospinal irradiation (CSI) after prior cranial radiation may be offered to carefully selected patients but is not without risks, particularly if critical normal structures have already been treated to tolerance doses. Limited clinical data suggest acceptable safety and reasonable efficacy of salvage CSI in children with recurrent, previously-irradiated ependymoma [1], recurrent medulloblastoma after prior CSI [2], and in other histologies with neuroaxis dissemination after prior focal cranial radiation [3]. In standard CSI technique with opposed lateral cranial fields, blocking previously treated critical normal structures will also block large volumes of target brain tissue lateral to these structures, which leads to the possibility of tumor reseeding in these areas and could compromise the efficacy of treatment intended to treat the entire neuroaxis. Proton therapy is a modality of radiation therapy which has the physical advantage of a defined stopping point, called the Bragg peak, with sparing of tissues beyond that point due to the absence of exit dose [4]. We present a simple technique in proton CSI to shield critical normal structures from additional radiation while minimizing the volume of underdosed adjacent brain.

## Methods

### Patient cases

**Figure 1:**
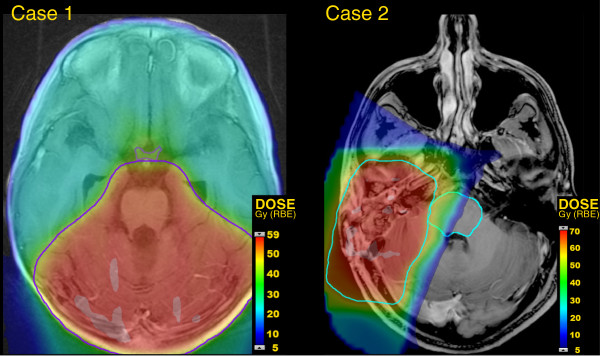
**Colorwash dose distributions for prior radiation therapy are shown.** Case 1, on the left, received treatment for medulloblastoma, 23.4 Gy (RBE) to the neuroaxis and a boost to the posterior fossa to 55.8 Gy (RBE). The 46 Gy (RBE) isodose line (purple) encroached upon the optic chiasm (purple). Case 2, on the right, received treatment for a grade III posterior fossa meningioma to 70.2 Gy (RBE). The 62 Gy (RBE) isodose line (cyan) abutted the lateral surface of the brainstem (cyan).

The authors were confronted by two clinical cases. In both cases, written informed consent was obtained from the patient or parent for publication of this report and accompanying images. The first case was a 9 year old boy who developed multilevel leptomeningeal recurrence 21 months after 23.4 Gy (RBE) CSI for a standard risk medulloblastoma with a posterior fossa boost to 55.8 Gy (RBE). Review of the prior radiation showed the 46 Gy (RBE) isodose line encroaching on the optic chiasm (Figure 1). After a good response to initial chemotherapy, salvage proton CSI to 36 Gy (RBE) was planned, followed by a boost to 54 Gy (RBE), provided the optic chiasm could be spared from additional radiation dose. Dose to the brainstem was not attenuated, based on clinical data [1] on the relative safety of posterior fossa reirradiation, despite high cumulative doses to the brainstem.

The second case was a 37 year old man who developed multilevel leptomeningeal recurrence 5 months after postoperative proton therapy to 70.2 Gy (RBE) for a posterior fossa grade 3 meningioma. In the prior radiation plan, the 62 Gy (RBE) isodose line abutted the surface of the brainstem (Figure 1). Salvage proton CSI to 36 Gy (RBE) was planned, followed by a boost to 54 Gy (RBE), provided the previously treated right lateral surface of the brainstem could be spared from further radiation. In this case, the treating physician felt that the short interval from prior radiation therapy did not allow for any additional radiation dose to be delivered to the portion of the brainstem which had previously received 62 Gy (RBE), and the cumulative surface dose to the brainstem should be limited to 62 Gy (RBE).

### Delineation of avoidance structures

For both cases, the prior radiation treatment plans were coregistered to the new treatment planning simulation CT so that the prior delivered cranial radiation dose could be understood in relation to the new treatment planning scan, and a composite dose plan could be created. The organs at risk (OARs) were delineated using a coregistered treatment planning magnetic resonance image. For the first case, the treating physician (JB) delineated the optic chiasm and created a planning organ at risk volume (PRV) composed of the optic chiasm plus 8 mm, intending that the 50% isodose line of the retreatment plan should abut this avoidance structure. For the second case, the treating physician (MM) used the coregistered prior treatment plan to delineate the lateral length of the brainstem which had previously received 62 Gy (RBE) and used an anisotropic expansion of 1 cm laterally and 3 mm in the anterior to posterior and cranio-caudal direction around this portion of the brainstem to define the PRV, intending that the 50% isodose line of the retreatment plan should abut this avoidance structure.

The difference in PRV definition between the two cases reflected the individual best clinical judgment of the physicians involved, and consideration of individual treatment goals and uncertainties in the region of the inverse aperture. In the second case, recurrent tumor in the posterior fossa was in much closer proximity to the OAR, prompting a smaller PRV expansion in the anterior posterior direction, where PRV avoidance was provided by the aperture edge and uncertainties were limited to potential organ motion of the OAR and patient setup uncertainties. However in the lateral direction, OAR avoidance was dependent on the reliability of distal proton beam blocking. In addition to lateral patient setup uncertainties, potential daily variation in tissue heterogeneity in the proton beam path (in the second case including the mastoid air cells) was considered in creating a larger PRV expansion to provide an additional safety margin around the OAR.

### Proton treatment planning and delivery

Patients were immobilized in the supine position with a custom alpha-cradle and thermoplastic head mask, acquiring a treatment planning CT scan with 2 mm slice thickness from the top of the head to the proximal femurs. We have previously described our technique for supine proton CSI using a custom carbon-fiber table [5]. At our institution, patient positioning is accomplished on a robotic patient positioner with six degrees of freedom. Orthogonal kilovoltage image sets are taken every day for every treatment field, using bony anatomic landmarks as registration points to calculate required shifts to duplicate the patient position from the time of simulation. On-line verification of patient positioning is made by a physician prior to treatment of each field.

**Figure 2:**
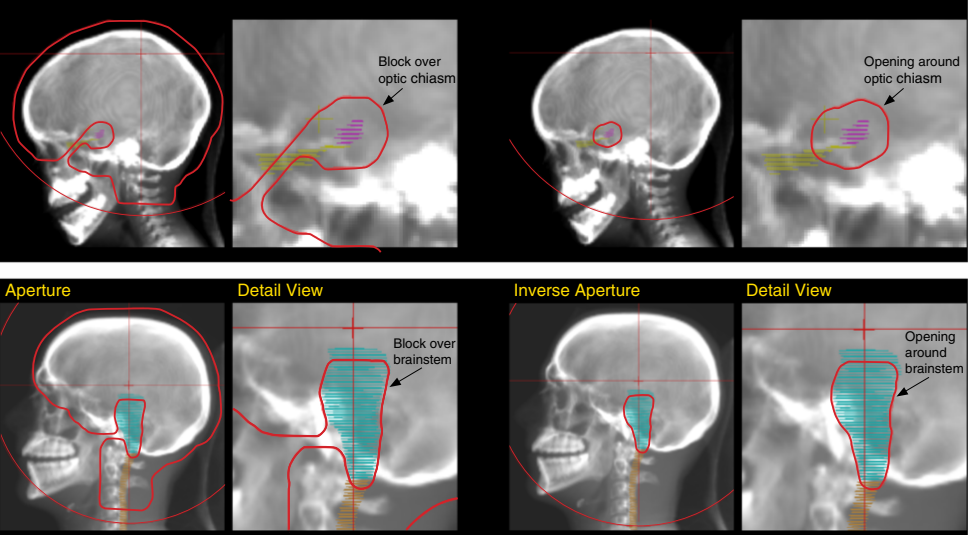
**Apertures shapes created for salvage craniospinal irradiation.** On the top row, for case 1, the whole brain apertures with a small block over the optic chiasm are shown on the left, and on the right, the inverse aperture to irradiate the target lateral to the optic chiasm. On the bottom row, for case 2, the whole brain apertures with a block over the brainstem are shown on the left, and on the right, the inverse aperture to irradiate the target lateral to the previously irradiated brainstem surface. Aperture outlines have been enhanced for visibility.

To treat the cranial portion of the target, right and left lateral cranial apertures were generated to treat the whole brain. A small block was extended from the closest edge of the aperture to block the previously delineated avoidance structure of the optic chiasm or brainstem (Figure 2). The right and the left lateral field were to be treated daily, with each of these fields delivering 50% of the fractional dose. These fields blocked the PRV and created plan dosimetry very similar to two lateral photon fields with a custom block over the PRV, leaving a dose shadow lateral to the blocked PRV.

To fill in these dose shadows and treat more of the target brain tissue lateral to the PRV, an inverse aperture was created. Taking the original aperture shape, an auto-contouring feature was used so that the area originally delineated as a block over the PRV was instead reversed as the aperture opening. The original aperture was now made inverse: the open cranial areas were now blocked and the previously blocked area over the PRV was now open. These inverse apertures were also both treated daily and each delivered 100% of the fractional dose, irradiating the target volume lateral to the structure of concern. The energy or range of the proton beam through the inverse apertures were selected so that the distal 50% isodose line of the beam through the right side would abut the right edge of the previously defined PRV and similarly for the beam through the left inverse aperture to abut the left edge of the PRV.

The first aperture sets with blocks and the inverse apertures shared the same isocenter for patient setup and created a radial matchline as the block edge of the first aperture was the field edge of the inverse aperture. To address the treatment dosimetry and delivery uncertainties created by this radial matchline, the inverse aperture opening around the PRV was made 1 mm smaller than the aperture block over the PRV, deliberately introducing a 1 mm gap at the margin of the abutting open and blocked fields. The selection of 1 mm was made *a posteriori* in the same way that some practitioners introduce a small gap between abutting cranial and spinal fields in photon craniospinal irradiation.

### Comparative treatment plans

For comparison to the proton treatment plan, the same CT and structure set were used to generate a conventional photon plan and an IMRT treatment plan addressing the cranial portion of the target with avoidance of the previously developed PRVs. To compare the intracranial target coverage, a planning target volume (PTV) was created for evaluation (cranial PTV 36 Gy) consisting of the intracranial target volume minus the PRV. The conventional photon plan utilized two opposed lateral cranial fields with 6 MV photons and a custom cerrobend block over the PRV on both fields to achieve sparing of the OAR. The IMRT treatment plan utilized 9 coplanar fields with the gantry at 0, 30, 60, 125, 160, 200, 230, 300, and 300 degrees. Priority during IMRT optimization was given to PRV avoidance so that the dose profile to the PRV and maximum dose would attempt to match the proton treatment plan, while maximizing the coverage of the cranial PTV 36 Gy.

## Results

**Figure 3:**
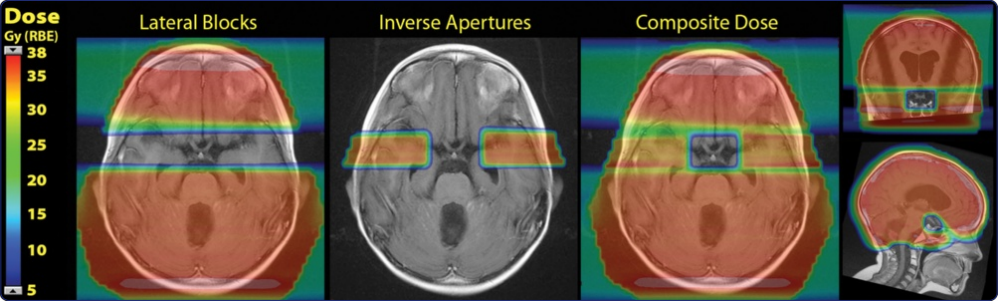
**Colorwash dose distributions for salvage craniospinal irradiation for case 1.** On the left, the dose from the lateral fields with blocks, and in the center, the dose from the lateral inverse apertures. On the right, the composite dose distribution in transverse, coronal and sagittal planes showing the zone of dose avoidance around the previously treated optic chiasm.

**Figure 4:**
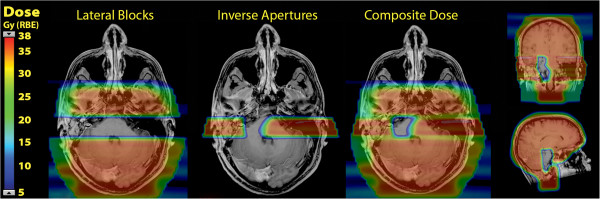
**Colorwash dose distributions for salvage craniospinal irradiation for case 2.** On the left, the dose from the lateral fields with blocks, and in the center, the dose from the lateral inverse apertures. On the right, the composite dose distribution in transverse, coronal and sagittal planes showing the zone of dose avoidance around the previously treated surface of the brainstem.

**Figure 5:**
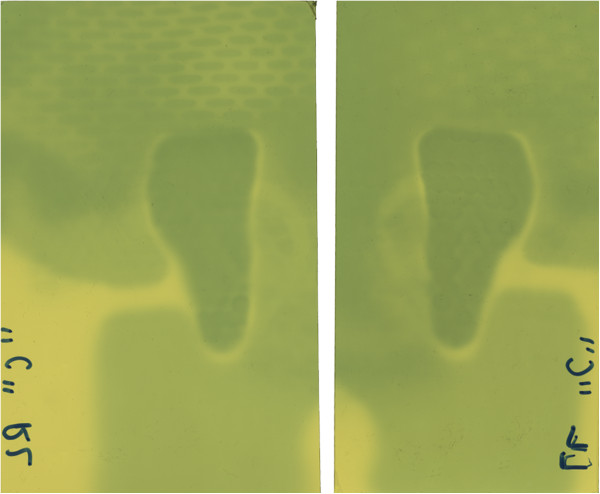
**Film dosimetry from patient case 2 is reproduced, showing the dose exposure after irradiation through both the lateral blocked field and inverse aperture field.** Film dosimetry was taken at the first day of treatment to verify the small radial gap between fields and the absence of dose overlap.

Salvage proton craniospinal treatment plans were developed and successfully implemented to provide sparing of previously irradiated critical normal structures while treating target brain lateral to these structures. The dose colorwash for the lateral fields with blocks, the inverse apertures, and the composite dose are shown for each case in Figures 3 and 4. The clinical setup was assessed by the treating physician to verify light fields through the open and inverse apertures on the patient immobilization mask, confirming no overlap of fields. On the first day of treatment, film dosimetry was performed to assess the junction of the open and inverse cranial apertures to ensure no dose overlap. An example of the film dosimetry from the second case is shown in Figure 5. After initial follow-up of nine months from the completion of reirradiation, both patients are alive and maintain disease control with no central nervous system toxicities.

**Figure 6:**
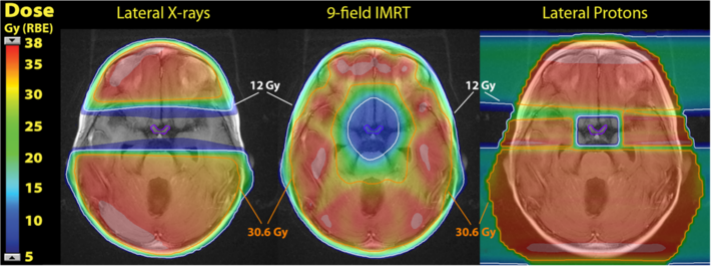
**Comparative treatment plans for case 1.** The dose distribution is shown in colorwash and the 12 Gy (33.3%) isodose line is delineated in white, while the 30.6 Gy (85%) isodose line is delineated in orange.

**Figure 7:**
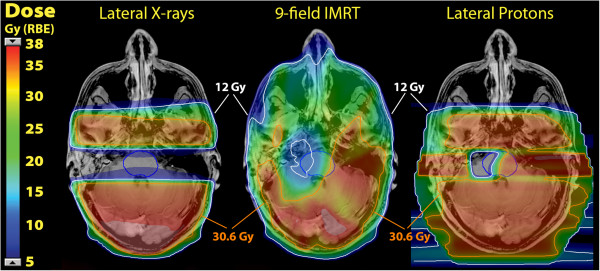
**Comparative treatment plans for case 2.** The dose distribution is shown in colorwash and the 12 Gy (33.3%) isodose line is delineated in white, while the 30.6 Gy (85%) isodose line is delineated in orange.

**Figure 8:**
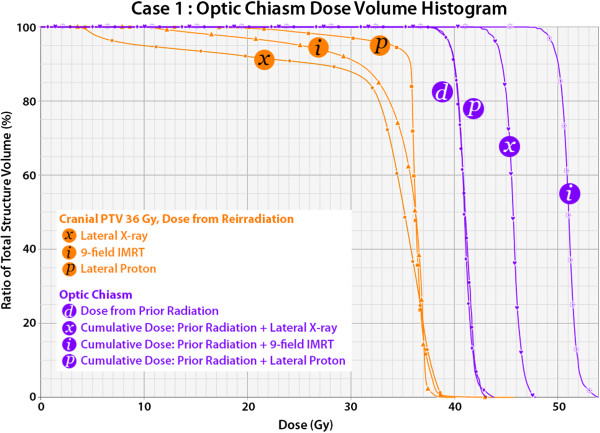
**Dose volume histogram for case 1.** The dose delivered in reirradiation to the cranial PTV 36 Gy is compared for three treatment options: lateral X-ray fields with blocks, a 9-field coplanar intensity modulated radiation (IMRT) plan, and a lateral proton fields with blocks and inverse apertures. The lateral proton plan provides improved coverage of the cranial PTV 36 Gy. The dose to the optic chiasm from the initial radiation treatment plan is shown, labeled “d”, while the cumulative dose to the optic chiasm from prior radiation and reirradiation is compared by the same three treatment plans. The lateral proton plan incurred virtually no additional dose to the optic chiasm, and the cumulative dose was less than with the lateral X-ray plan or the IMRT plan.

**Figure 9:**
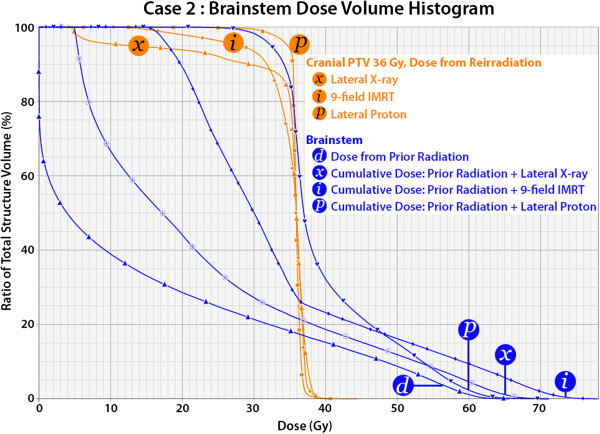
**Dose volume histogram for case 2.** The dose delivered in reirradiation to the cranial PTV 36 Gy is compared for three treatment options: lateral X-ray fields with blocks, a 9-field coplanar intensity modulated radiation (IMRT) plan, and a lateral proton fields with blocks and inverse apertures. The lateral proton plan provides improved coverage of the cranial PTV 36 Gy. The dose to the brainstem from the initial radiation treatment plan is shown, labeled “d”, while the cumulative dose to the brainstem from prior radiation and reirradiation is compared by the same three treatment plans. The lateral proton plan incurred minimal additional dose to the brainstem, and the cumulative dose was less than with the lateral X-ray plan or the IMRT plan.

Comparative treatment plans are demonstrated for each case in Figures 6 and 7. Using lateral photon cranial fields with custom cerrobend blocking, a dose distribution nearly identical to that with lateral proton cranial fields with blocking of the OAR is achieved, both blocking volumes of target brain tissue lateral to the OAR. Using a 9 field coplanar intensity modulated photon radiation treatment (IMRT) plan, a zone of central dose attenuation was created around the PRV, although due to the physical properties of x-rays, it was not possible to achieve a zone of zero radiation at the OAR with multi-field IMRT due to exit dose from multiple beam angles. Quantitative comparison of the treatment plans may be made in the dose volume histograms in Figures 8 and 9, while Table 1 provides comparison of target volume coverage and dose to the OAR for the comparative treatment plans. Compared to both the lateral X-ray plan and the 9-field coplanar IMRT plan, the lateral proton reirradiation technique provided improved coverage of the cranial PTV 36 Gy while adding the least additional radiation dose to the previously irradiated OAR of concern.

**Table 1:** Dosimetric evaluation of treatment plans

**Case 1**				
**Cranial PTV 36 Gy, Dose from Reirradiation**				
		**X-ray**	**IMRT**	**Proton**
PTV covered by 65% IDL (23.4 Gy)		91.0%	95.8%	99.1%
PTV covered by 90% IDL (32.4 Gy)		82.1%	86.1%	96.1%
PTV covered by 95% IDL (34.2 Gy)		64.2%	76.8%	94.8%
Minimum Dose to PTV		3.2 Gy	9.9 Gy	0.9 Gy
Mean Dose to PTV		32.9 Gy	34.6 Gy	36.0 Gy
Dose covering 95% of PTV		9.8 Gy	25.2 Gy	34.0 Gy
**Cumulative Dose to Optic Chiasm from Both Courses of Radiation**				
	**Prior RT**	**X-ray**	**IMRT**	**Proton**
Mean dose	41.0 Gy	45.6 Gy	51.0 Gy	41.0 Gy
Maximum point dose	43.2 Gy	47.9 Gy	54.0 Gy	43.9 Gy
**Case 2**				
**Cranial PTV 36 Gy, Dose from Reirradiation**				
		**X-ray**	**IMRT**	**Proton**
PTV covered by 65% IDL (23.4 Gy)		92.9%	96.9%	99.9%
PTV covered by 90% IDL (32.4 Gy)		88.5%	87.9%	98.1%
PTV covered by 95% IDL (34.2 Gy)		85.7%	76.6%	96.1%
Minimum Dose to PTV		3.6 Gy	11.5 Gy	6.5 Gy
Mean Dose to PTV		33.9 Gy	34.8 Gy	35.8 Gy
Dose covering 95% of PTV		12.8 Gy	28.1 Gy	34.7 Gy
**Cumulative Dose to Brainstem from Both Courses of Radiation**				
	**Prior RT**	**X-ray**	**IMRT**	**Proton**
Dose to 0.5 cc	59.7 Gy	64.4 Gy	70.9 Gy	61.2 Gy
Dose to 1 cc	57.8 Gy	62.1 Gy	68.6 Gy	59.3 Gy
Maximum point dose	65.6 Gy	71.2 Gy	78.1 Gy	71.2 Gy

## Discussion

While cranial reirradiation may be clinically appropriate in some cases, cumulative radiation dose to critical normal structures remains a practical concern. Clinical experience and animal data demonstrate that normal brain tissues, including the brainstem and spinal cord, exhibit substantial recovery from prior radiation over time [6]. The relatively low risk of radiation necrosis and myelopathy seen in patients reirradiated for brainstem gliomas, supratentorial gliomas, and metastatic brain disease may be explained by the relatively short median survival of these patients [7]. Conversely, in patients with a good prognosis or longer anticipated survival, central nervous system toxicity may be seen, even in patients with a long latency between initial cranial radiation and retreatment [8]. Practitioners and patients must deliberate carefully on the risk benefit ratio of further radiation therapy, define risk tolerance and acceptable and unacceptable toxicities, and within those parameters make best estimates of acceptable additional dose tolerance of critical normal structures.

Dose attenuation to the previously irradiated upper cervical spinal cord during salvage CSI for ependymoma was described by Merchant et al. [1] by blocking this region after delivery of an additional 16.2 Gy, limiting the cumulative dose to 55.8 Gy. In the two cases presented here, critical normal structures were judged to be at or near maximum acceptable cumulative dose. In this setting, if reirradiation is offered, blocking these structures on lateral fields will exclude a large volume of target brain tissue in order to shield the normal structure. Our technique provides a relatively simple method to provide a region of central dose sparing while minimizing the volume of target brain tissue that is excluded from reirradiation. In both patient cases, sparing the structure of concern required only two additional sets of apertures and compensators, and since additional treatment angles were not required, only modestly impacted the treatment duration.

Alternatives to this approach include the use of intensity modulated radiation therapy or volumetric modulated arc therapy to create a zone of dose attenuation around the structure of concern, similar to hippocampal avoidance during whole brain radiation [9]. Wei and colleagues [3] reported a series of 6 patients with prior cranial irradiation who received salvage craniospinal irradiation to 36 Gy using IMRT to attenuate dose to the previously irradiated brain tissue. No dosimetric data were provided to evaluate the degree of sparing of previously irradiated tissues or to provide comparison to alternative techniques. Compared to photon techniques, proton therapy has dosimetric advantages in the spinal portion of craniospinal irradiation [10] which eliminates acute radiotherapy toxicity that would be expected from radiation dose that would otherwise exit into the viscera anterior to the spine, and is expected to translate to reductions in late toxicity of therapy [11]. Using our technique of blocking structures and then “plugging” dose in lateral to those structures through inverse apertures, proton CSI can also achieve areas of central dose avoidance, and could be modified to produce regions of dose attenuation if desired, for example to treat previously irradiated tissues at a lower fractional dose than radiation-naïve volumes. It is anticipated that the implementation of intensity modulated proton therapy will allow for generation of similar regions of dose avoidance or attenuation without the requirement of patient specific hardware used in this technique.

Some may question the value of employing proton therapy, which is presently more expensive than photon-based radiation techniques, for salvage craniospinal irradiation. In our opinion, there are simply insufficient clinical data to address the question of the cost:benefit ratio of employing more conformal radiation techniques in this setting. Although clinical data continues to emerge from multiple institutions, reirradiation remains a highly individualized clinical challenge. In the judgment of the treating physicians, both of the cases presented here were anticipated to be potential long-term survivors and to meet the constraints placed on the OARs, large volumes of the cranial PTV 36 Gy would have been untreated or underdosed with other techniques, potentially increasing the risk of CNS failure.

Limitations of this technique include the inability to create a “floating” block, and a minimum field size for the inverse aperture in order to preserve the desired Bragg peak and accurately model proton dosimetry [12]. Additionally, extra attention to patient setup and immobilization is required due to the risk of intrafractional motion and dose overlap during treatment of the inverse aperture. We chose to minimize this risk by creating the inverse aperture 1 mm smaller than the lateral block and verified the setup using film dosimetry to ensure no dose overlap occurred. An alternative or adjunct to utilizing a small radial gap would be to feather the radial field junction by developing multiple sets of blocked and inverse apertures of varying sizes to move the location of field abutment during the treatment course in the same way that cranial and spine field junctions are feathered during a CSI course.

## Conclusions

Proton craniospinal irradiation can be adapted to provide dose sparing of previously treated critical normal structures. In this technique, lateral proton cranial field apertures block the structure of concern while inverse apertures treat the portion of target brain lateral to the organ at risk that would otherwise be underdosed. This technique provided more complete coverage of the intracranial target volume than could be achieved using lateral photon fields with blocks or with IMRT and may extend the option of reirradition to patients otherwise deemed ineligible for further radiotherapy due to prior dose to critical normal structures.

## Competing interests

The authors declare that they have no competing interests.

## Authors’ contributions

MM jointly developed the treatment planning concept, drafted the manuscript, and prepared the figures. MW and JS implemented the technique. JB jointly developed the treatment planning concept. All authors read and approved the final manuscript.
